# *Legionella pneumophila* Type IV Effectors YlfA and YlfB Are SNARE-Like Proteins that Form Homo- and Heteromeric Complexes and Enhance the Efficiency of Vacuole Remodeling

**DOI:** 10.1371/journal.pone.0159698

**Published:** 2016-07-26

**Authors:** Eva M. Campodonico, Craig R. Roy, Shira Ninio

**Affiliations:** 1 Section of Microbial Pathogenesis, Yale University School of Medicine, New Haven, Connecticut, United States of America; 2 The Yigal Allon Kinneret Limnological Laboratory, Israel Oceanographic and Limnological Research, Migdal, Israel; Purdue University, UNITED STATES

## Abstract

*Legionella pneumophila* is a Gram-negative bacterium that can colonize both freshwater protozoa and human alveolar macrophages, the latter infection resulting in Legionnaires’ disease. The intracellular lifecycle of *L*. *pneumophila* requires extensive manipulation of its host cell, which is carried out by effector proteins that are translocated into the host cell through the Dot/Icm type IV secretion system. This study focuses on a pair of highly similar type IV substrates called YlfA/LegC7 and YlfB/LegC2 that were initially identified in a screen for proteins that cause growth inhibition in yeast. Analysis of truncation mutants revealed that the hydrophobic residues in the Ylf amino termini were required for localization of each protein to the membranes of host cells. Central and carboxy terminal coiled coil domains were found to mediate binding of YlfA and YlfB to themselves and to each other. *In vivo*, a *ΔylfA ΔylfB* double mutant strain of *L*. *pneumophila* was shown to be defective in establishing a vacuole that supports bacterial replication. This phenotype was subsequently correlated with a decrease in the association of endoplasmic reticulum (ER)-derived vesicles with vacuoles containing *ΔylfA ΔylfB* mutant bacteria. These data suggest that the Ylf proteins are membrane-associated effectors that enhance remodeling of the *L*. *pneumophila* -containing vacuole by promoting association and possibly fusion of ER-derived membrane vesicles with the bacterial compartment.

## Introduction

In nature, the facultative intracellular bacterium *Legionella pneumophila* establishes a replicative niche within numerous protozoan species found in freshwater ponds, lakes and streams [1]. *L*. *pneumophila* can also infect and replicate within the alveolar macrophages of the human lung, gaining access to these cells following inhalation of aerosols from contaminated man-made reservoirs, such as cooling towers and air conditioning systems [2]. The resulting respiratory infection is known as Legionnaires’ disease, a potentially fatal pneumonia in humans [3,4].

Despite the evolutionary divergence of *Legionella*’s mammalian and protozoan hosts, it has been well established that the same sequence of intracellular trafficking events occurs following *L*. *pneumophila* infection of each cell type. *L*. *pneumophila* inhibits fusion of the vacuole in which it resides with degradative endosomes and lysosomes within the first minutes of infection [5,6]. Concurrently, mitochondria and membrane vesicles derived from the early secretory pathway are recruited to the *L*. *pneumophila* -containing vacuole (LCV) [7]. Subsequent fusion of endoplasmic reticulum (ER)-derived vesicles with the LCV generates an ER-like, ribosome-decorated compartment that supports bacterial replication [8–10]. Roughly 24 hours later, following multiple rounds of bacterial replication, this compartment and the host cell lyse, releasing *L*. *pneumophila* to infect surrounding cells.

A type IV secretion system called Dot/Icm is essential for *L*. *pneumophila* virulence [11–13]. The coordinate action of numerous effector proteins that are translocated into the host cell by the Dot/Icm apparatus dictates intracellular transport of the LCV [14–18]. Although the biochemical activities of most Dot/Icm effector proteins remain unknown, several effectors of known function have been shown to target eukaryotic proteins involved in membrane transport (reviewed [19,20]).

The recruitment of ER derived vesicles to the LCV is one of the central steps in the biogenesis of an organelle that supports *L*. *pneumophila* replication, and a number of effector proteins have been shown to be involved in this step. The first effector to be described was the protein RalF, which is a guanine nucleotide exchange factors (GEF) that activates host Arf on the LCV by stimulating GDP/GTP exchange [21]. The small GTPase Rab1, which regulates membrane trafficking events between the ER and the Golgi, is targeted by at least five different *L*. *pneumophila* effectors—DrrA, AnkX, SidD, Lem3 and LepB. These effectors function to coordinate the activation, and deactivation of Rab1 on the LCV, facilitating the recruitment of ER derived vesicles to the LCV (reviewed [22]). Previous studies indicate that the host SNARE protein Sec22b localizes to the LCV, and that the function of this protein is important for efficient bacterial replication [23,24]. However, the absence of Membrin on the LCV [23,24], which is the cognate t-SNARE that binds Sec22b, suggests that *L*. *pneumophila* encodes Dot/Icm substrates that enhance membrane fusion with the LCV. The effector DrrA is one such example. DrrA activates the Rab1 GTPase on plasma membrane-derived organelles facilitating the tethering of ER-derived vesicles with the plasma membrane derived organelle, resulting in vesicle fusion through the pairing of Sec22b with non-cognate syntaxin proteins on the plasma membrane [25,26]. It is likely that additional *L*. *pneumophila* effectors also function to facilitate the fusion of ER-derived vesicles with the LCV, since functional redundancy of effectors is anticipated in crucial stages of the *L*. *pneumophila* lifecycle. Other effector proteins may stimulate membrane fusion either in concert with Sec22b, or in a manner independent of host cell proteins. Here we investigate a pair of Dot/Icm effectors, YlfA and YlfB, which may play a role in membrane fusion events that contribute to the biogenesis of a compartment that supports *L*. *pneumophila* replication.

The SNARE—like Dot/Icm effectors YlfA and YlfB were identified in a screen for proteins that cause growth inhibition in yeast [27]. YlfA and YlfB share significant sequence homology (41% identical, 62% similar), and these proteins are predicted to contain a hydrophobic region and coiled coil domains based on primary amino acid sequence analysis [27,28]. Though these motifs do not indicate a specific function, they generally suggest that upon translocation, the Ylfs could localize to host membranes and participate in protein-protein interactions. Indeed, endogenous YlfA protein is observed on the ER-derived replicative vacuole and on punctate structures throughout the host cell at late stages of infection. Ectopically produced YlfA localizes to early secretory organelles, and localization is driven by the N-terminal hydrophobic domain of the protein [27]. Interestingly, there is significant amino acid similarity between YlfA and the *Chlamydophila pneumoniae* protein IncA, which localizes to the inclusion membrane of this obligate intracellular pathogen [29,30]. IncA is required for homotypic fusion of isolated *Chlamydiae* inclusions [31]. Recent work has shown that *C*. *trachomatis* IncA interacts with numerous SNARE proteins [32], and that the coiled-coil domains of IncA are required for driving homotypic membrane fusion [33].

In this study, we analyze the hydrophobic regions and putative coiled coil domains of YlfA and YlfB, and demonstrate that these motifs mediate association with host membrane, and formation of protein complexes, respectively. In addition, we investigate the role of the Ylfs in the generation of the bacterial compartment, and show that these proteins contribute to the efficient acquisition of ER-derived membrane by the LCV.

## Results

### A Hydrophobic Region in the Ylf Proteins Mediates Membrane Localization

**Fig 1:**
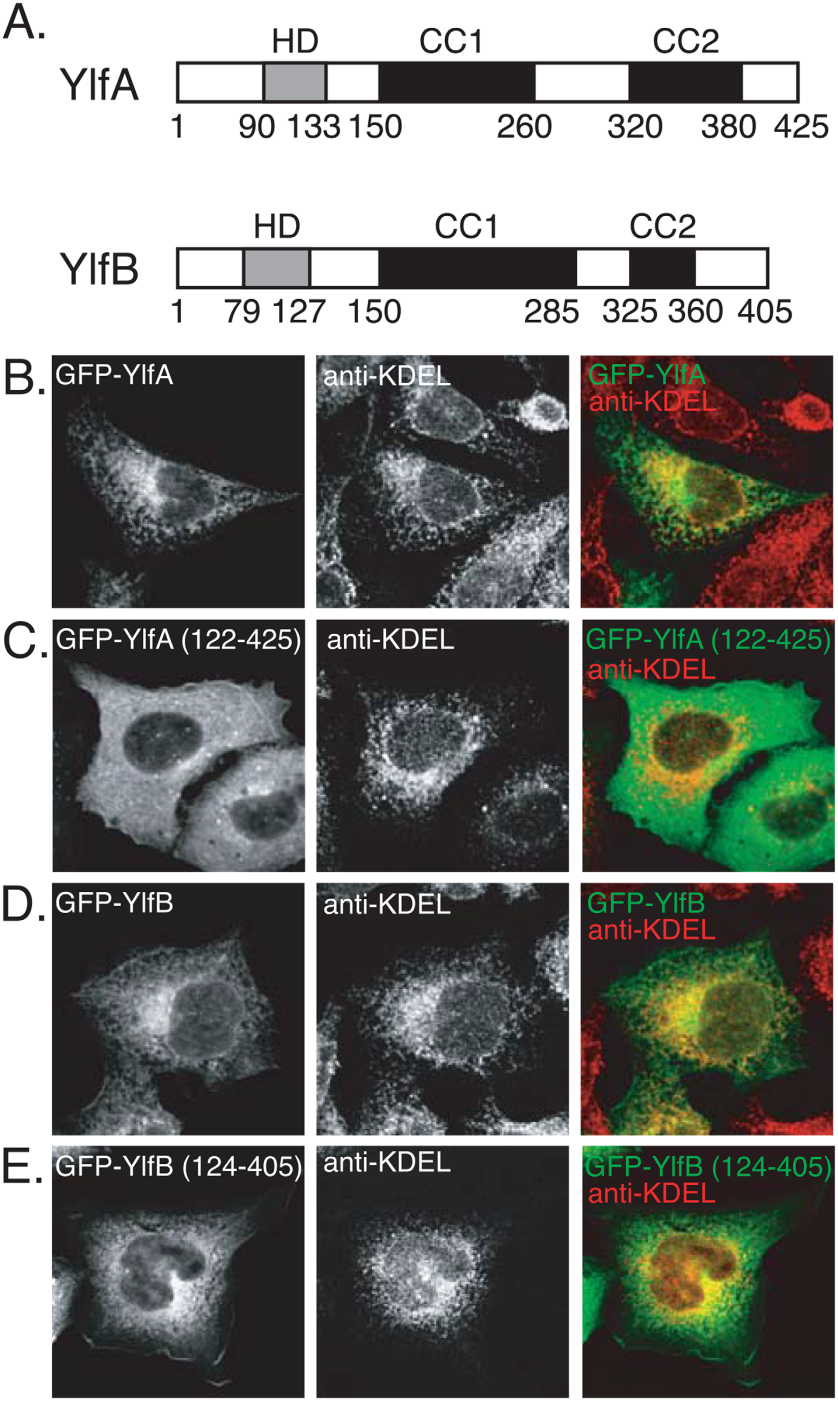
Amino terminal hydrophobic residues mediate colocalization of YlfA and YlfB with eukaryotic membrane networks. (A) Predicted structural motifs of YlfA and YlfB. The amino terminal hydrophobic domains (HD), in grey, were identified by analysis of the YlfA and YlfB amino acid sequences using Kyte-Doolittle [48], TMBase25 [49] and TMHMM2.0 [50] algorithms. Regions predicted to form coiled coil domains (CC), in black, were defined using the COILS server. (B-E) Cellular localization of GFP-tagged Ylf proteins. CHO cells were transfected with plasmids encoding amino-terminal GFP-tagged proteins: full length YlfA (B), YlfA 122–425 (C), full length YlfB (D), and YlfB 121–405 (E). 18 h after transfection, cells were fixed and endogenous KDEL-containing proteins were stained with a mouse anti-KDEL antibody, followed by an AlexaFluor 594-conjugated anti-mouse secondary antibody. Grey scale images of confocal sections show the localization of GFP-tagged proteins and KDEL-containing proteins demarcating the early secretory pathway. Merged color images show GFP-Ylf fusion proteins (green) and anti-KDEL staining (red).

Analysis of the Ylf sequences revealed regions of amino acid residues with sufficient hydrophobic potential to serve as transmembrane domains. A region in YlfA between amino acids 90 and 133 was predicted to form a single transmembrane domain (Fig 1A). A potential membrane-spanning region was located between amino acids 79 and 127 of YlfB. TMHMM2.0 identified two putative transmembrane helices between YlfB residues 79–101, and 105–127. The central and carboxy terminal residues of YlfA and YlfB are predicted to form two regions of coiled coils, which have been labeled CC1 and CC2 (Fig 1A).

To investigate whether these hydrophobic regions influence the localization of the Ylf proteins, CHO cells were transfected with plasmids producing either full-length or truncated GFP-Ylf fusion proteins. The amino terminus of YlfA was required for a reticulate localization pattern upon ectopic production in mammalian cells. Staining of transfected CHO cells with an antibody that recognizes the ER retrieval sequence KDEL revealed that GFP-YlfA localized to a membrane network of the early secretory pathway (Fig 1B). In contrast, GFP-YlfA 122–425, was redistributed to the cell cytosol (Fig 1C). These data indicate that the localization of YlfA with endomembrane networks is significantly disrupted by truncation of the hydrophobic region of this protein, suggesting that these residues mediate interactions with host membranes. Furthermore, our data suggests that a short stretch of 11 hydrophobic amino acids, too short to traverse a membrane bilayer, is not sufficient for driving the membrane targeting of GFP-YlfA 122–425 in eukaryotic cells. Similar to what was observed with GFP-YlfA, the GFP-YlfB protein localized largely to a reticulate network in the cell (Fig 1D). Anti-KDEL staining of transfected cells indicated that ectopically produced GFP-YlfB colocalized extensively with the endoplasmatic reticulum. As was observed for YlfA, a truncation derivative lacking the hydrophobic region, GFP-YlfB 124–405, showed some redistribution to the cytosol (Fig 1E), though this mutant did retain a moderate level of perinuclear colocalization with KDEL-positive structures. Both full-length and truncated GFP-YlfB proteins were detected on KDEL-negative structures at the plasma membrane of transfected cells. RFP-YlfB 1–298, which lacks the predicted CC2 domain but retains the CC1 domain, was not observed at the plasma membrane of transfected cells, however production of RFP- YlfB 301–405 (containing only the CC2 domain) resulted in a cytosolic distribution with some detectable plasma membrane localization (S1 Fig). These results demonstrate that the CC2 domain is required for localization of the YlfB protein to the plasma membrane and can also drive the membrane localization of the protein, albeit inefficiently. Expression of the YlfB 124–405 protein containing both the CC1 and CC2 domains results in more pronounced membrane localization (Fig 1E), suggesting that the CC1 region also contributes to the localization of YlfB to the plasma membrane but requires the presence of CC2 to do so.

**Fig 2:**
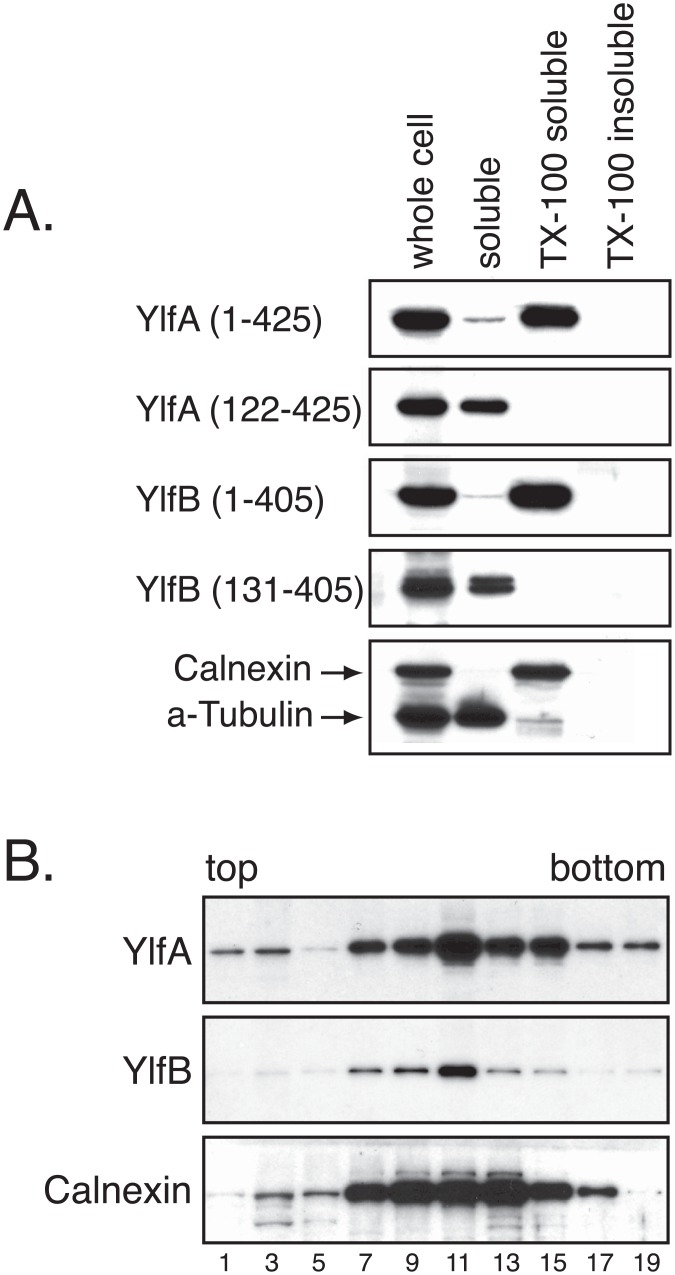
Amino terminal hydrophobic residues mediate association of YlfA and YlfB with eukaryotic lipid. HEK293T cells were transfected for 14 h with plasmids encoding full length (A and B) or truncation mutant Ylf proteins (A). A) Membrane-associated and soluble protein fractions were generated by centrifugation of whole cell lysate at 100,000 x g, followed by solubilization of membrane-associated proteins with 0.1% Triton X-100. The presence of Ylf protein in each subcellular fraction is detected by anti-YlfA and anti-YlfB immunoblot. To demonstrate effective fractionation of cell lysates, fractions were probed with antibodies against the transmembrane protein Calnexin and cytosolic alpha-Tubulin. B) Whole cell lysates were overlayed on a 4–26% continuous OptiPrep gradient and centrifuged at 100,000 x g for 3 h. The presence of Ylf protein in each odd fraction was detected by immunoblot using anti-YlfA or anti-YlfB antibodies. The sedimentation profile of membranes associated with the ER and early secretory pathway was revealed by anti-Calnexin immunoblot.

Fractionation studies using HEK293T cells producing either full-length or amino terminal truncation derivatives of the Ylf proteins were used to further investigate whether the hydrophobic regions mediate interactions with host membranes (Fig 2A). Consistent with the imaging data, the majority of full length YlfA was in a pellet fraction containing detergent-soluble host membrane proteins. YlfA protein lacking the amino terminal hydrophobic region fractionated with soluble components of the cell. Likewise, full length YlfB fractionated with detergent-soluble host membrane proteins. Despite the apparent localization of GFP-YlfB 124–405 with KDEL-positive membranes (Fig 1E), this truncation derivative of YlfB fractionated exclusively with soluble components of the cell. Ylf localization was also examined by density-gradient fractionation. The centrifugation parameters used retained soluble factors in the top fractions while allowing for the recovery of ER-derived membrane vesicles in the middle third of the gradient, as is indicated by the peak detection of the integral membrane resident ER protein Calnexin in fractions 11 and 13 (Fig 2B). Protein aggregates were found in the bottom fraction. Peak fractions containing YlfA and YlfB overlapped with fractions containing Calnexin, indicating that the Ylf proteins were associated with host membranes of the early secretory pathway (Fig 2B).

### YlfA and YlfB Form Homotypic and Heterotypic Complexes

**Fig 3:**
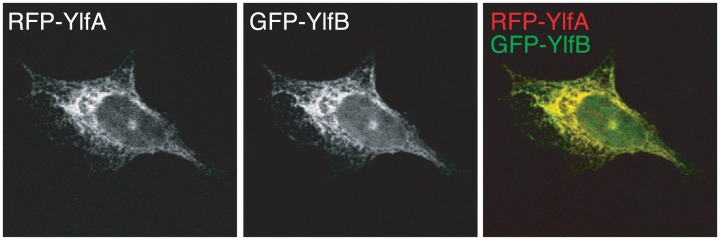
Co-production of YlfA and YlfB alters localization of YlfB. CHO cells were co-transfected for 18 h with plasmids encoding amino terminal RFP or GFP-tagged full length YlfA and YlfB. Cells were fixed in 2% PFA, mounted and imaged. Grey scale images show the localization of RFP and GFP-tagged proteins in confocal sections. Merged color images reveal colocalization of YlfA and YlfB.

YlfA and YlfB displayed similar, though not identical patterns of localization when ectopically produced separately in eukaryotic cells. However, during *L*. *pneumophila* infection the Ylfs should both be present within the cell. RFP-YlfA and GFP-YlfB were co-produced in cells to determine whether expression of one Ylf protein could influence the localization of the other (Fig 3). Under these conditions the reticulate localization pattern of both proteins was retained, however, GFP-YlfB was no longer detected at the cell periphery and colocalization of the two proteins appeared to be complete. This data suggest that YlfA and YlfB were capable of complex formation when coproduced in eukaryotic cells.

**Fig 4:**
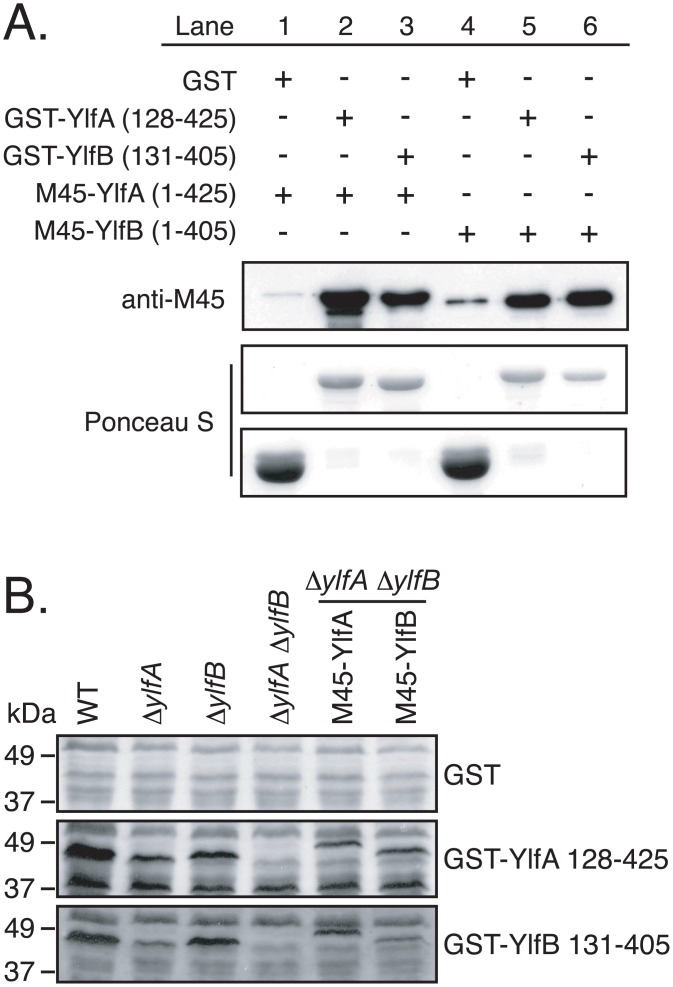
YlfA and YlfB bind homo- and heterotypically *in vitro*. A) Lysates from *E*. *coli* co-transformed with plasmids encoding GST and M45-tagged Ylfs were incubated with GS4B resin. Bound proteins were eluted with reduced glutathione and eluates were analyzed by anti-M45 epitope immunoblot. To assess the relative efficiency of binding and elution of GST and GST-Ylf fusion proteins, PVDF membranes were subsequently stained with Ponceau S. B) Whole cell lysates of stationary phase *L*. *pneumophila* were separated by SDS-PAGE and immobilized on PVDF. *L*. *pneumophila* strains include wild type (lane 1), in-frame deletion Δ*ylfA* and Δ*ylfB* mutants (lanes 2, 3), a double in-frame deletion Δ*ylfA* Δ*ylfB* mutant (lane 4), and the Δ*ylfA* Δ*ylfB* mutant harboring plasmids encoding M45-YlfA or M45-YlfB (lanes 5, 6.) Membranes were incubated with recombinant purified GST, GST-YlfA 128–425, or GST-YlfB 131–405 suspended at 20 μg/ml in 2% NFDM 0.1% Tween PBS overnight at 4°C. Bound GST or GST fusion protein was detected by anti-GST immunoblot.

To address whether YlfA and YlfB interact directly or if additional factors present in transfected cells were required for complex formation, Ylf binding was assessed by GST pull-down from *E*. *coli* producing both GST-Ylf and M45-Ylf fusion proteins (Fig 4A). Both M45-YlfA and M45-YlfB bound GST-YlfA and GST-YlfB at roughly equivalent levels, indicating that the Ylfs form homomeric and heteromeric complexes independent of eukaryotic factors.

To test whether other Dot/Icm-translocated proteins might interact with YlfA and YlfB, GST-Ylf fusion protein probes were used to test for interactions with proteins in whole cell *L*. *pneumophila* lysates (Fig 4B). Using GST-YlfA 128–425 or GST-YlfB 131–405 to probe immobilized lysates of wild type *L*. *pneumophila*, a doublet of binding partners was detected that migrated close to the 48 kDa marker. The molecular weights of each band were consistent with those of full length YlfA (47.5 kDa) and YlfB (46.5 kDa) proteins. Purified GST-Ylf probes and immobilized lysates derived from a series of Δ*ylfA* and Δ*ylfB* mutant and complemented strains were used to confirm that the interacting proteins in wild type cell lysates were YlfA and YlfB. In addition to the Ylf interactions, the YlfA probe showed binding to an approximately 40 kDa protein in wild type lysate. The appearance of this 40 kDa band in all other lysates, including the Δ*ylfA* Δ*ylfB* mutant, indicates that this was not a degradation product of YlfA or YlfB. Therefore, this band may represent an additional binding partner for YlfA, but the identity of this protein is unknown.

### The Predicted Coiled Coil Domains Are Sufficient to Form Dimeric and Trimeric Complexes of YlfA and YlfB

**Fig 5:**
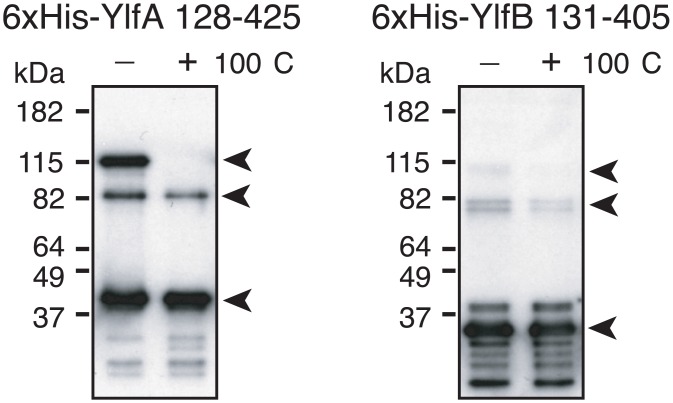
Purified YlfA and YlfB form high molecular weight complexes that are resistant to boiling. 6xHis-tagged YlfA 128–425 and YlfB 131–405 were applied to a Superdex 200 column. Peak fractions of each protein were combined with Laemmli buffer containing β-mercaptoethanol and a subset were heated for 10 min at 100°C. Protein complexes were detected by anti-YlfA and anti-YlfB immunoblot. Arrows indicate bands corresponding to monomeric, dimeric and trimeric species.

To characterize the Ylf complexes in more detail, purified YlfA and YlfB coiled coil domains were examined for the ability to form stable, higher-order complexes. Analysis of Ylf complexes using gel filtration was complicated by the tendency of these purified proteins to self-associate into soluble aggregates in purified solution. Therefore, the majority of the protein migrated in either the void volume or in fractions associated with a very large (500 kDa +) species that is unlikely to represent a physiologically relevant complex of Ylf protein (data not shown). However, immunoblot analysis of this peak fraction revealed a subpopulation of Ylf protein that associates in smaller, stable complexes. A peak fraction of His-YlfA 122–425 resolved into three prominent species in the absence of boiling. Bands at approximately 40, 80 and 120 kDa indicated the presence of monomeric, dimeric and trimeric population of YlfA 122–425 (Fig 5). When samples were subjected to 100°C, only the 120 kDa band was eliminated, indicating that His-YlfA 122–425 formed very stable protein dimers. Analysis of His-YlfB 131–405 was hindered by the tendency of this protein to degrade. However, a pattern of higher order complex formation similar to YlfA was observed, with bands corresponding to monomeric, dimeric and trimeric species in the absence of boiling. Again, only the trimer disappeared with boiling, suggesting that His-YlfB 131–405 also formed stable dimers in solution.

### YlfA and YlfB Are Required for the Efficient Remodeling and Establishment of the *L*. *pneumophila*—Containing Vacuole

It was shown that *L*. *pneumophila* mutant strains lacking either one or both *ylf* genes replicate comparably to wild type *L*. *pneumophila* in CFU-based intracellular replication assays [27]. Similar results have been obtained for numerous *L*. *pneumophila* mutants deficient in Dot/Icm substrates [34,35], a phenomenon that is in part due to a lack of sensitivity of the CFU-based assay to detect differences in the efficiency of vacuole transport. For this reason, *ylf* mutant *L*. *pneumophila* were examined using a more sensitive, single cell assay [36]. This assay measures the efficiency with which an internalized bacterium is able to remodel the plasma membrane-derived early phagosome in which it initially resides into an ER-derived organelle that permits replication.

**Fig 6:**
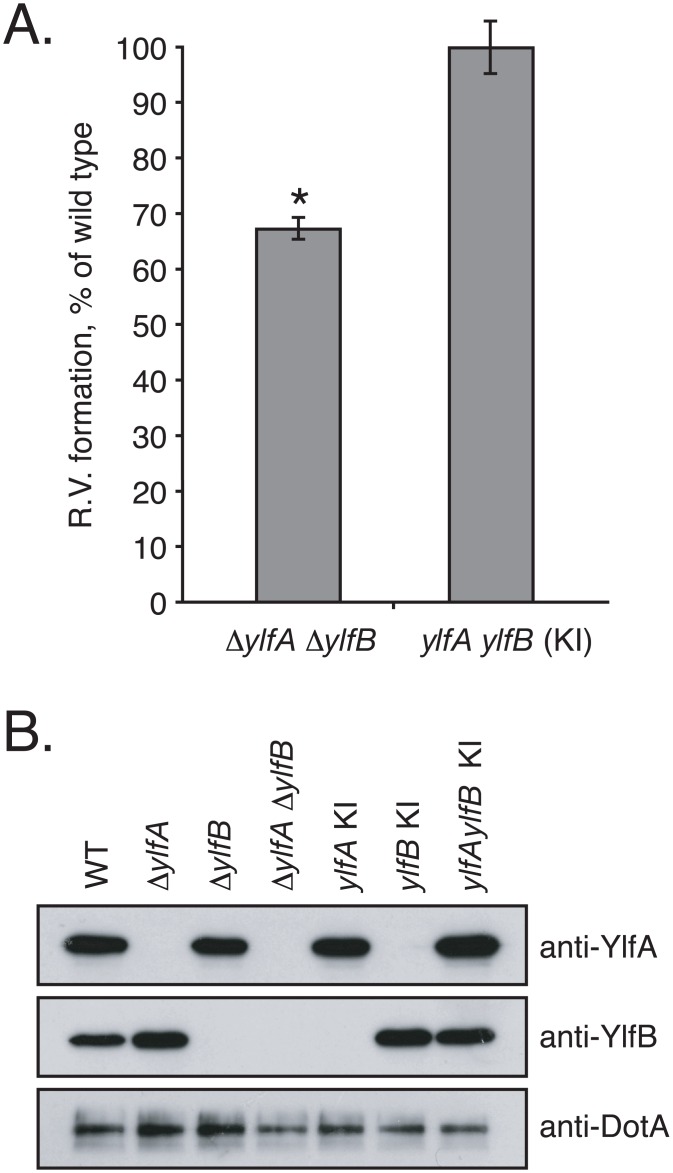
YlfA and YlfB are required for efficient formation of the *L*. *pneumophila*-containing vacuole. A) Bone marrow-derived macrophages derived from A/J mice were infected with wild type *L*. *pneumophila*, a Δ*ylfA* Δ*ylfB* mutant, and the Δ*ylfA* Δ*ylfB* mutant complemented by knock-in of both *ylf* ORFs onto the *L*. *pneumophila* chromosome. Duplicate samples were fixed at 2 and 10 h post-infection. The index of replicative vacuole formation was calculated by dividing the number of observed replicative vacuoles at 10 h by the total number of infected cells at 2 h. Due to variability in the overall efficiency of infection between individual experiments, the index for each strain is presented as a percent of wild type. Data represent the average of three independent experiments ± SD, n = 500 per time point, per experiment. Δ*ylfA* Δ*ylfB* mutant R.V. formation is significantly less efficient compared to wild type in an unpaired Student’s *t* test.*p<0.0001. B) Whole cell lysates were prepared from stationary phase broth cultures of wild type *L*. *pneumophila*, Δ*ylfA*, Δ*ylfB*, Δ*ylfA* Δ*ylfB*, and Δ*ylfA* Δ*ylfB* in which one or both *ylf* open reading frames have been reintroduced into the genome. YlfA and YlfB protein levels in these strains were detected by anti-YlfA and anti-YlfB immunoblot. Relative loading efficiency was monitored by anti-DotA immunoblot.

Consistent with the CFU-based assay, after 10 hours of infection in bone marrow-derived macrophages, the Δ*ylfA* Δ*ylfB* double mutant revealed a 30% decrease in the number of vacuoles supporting replication of the mutant strain compared with wild type *L*. *pneumophila* (Fig 6A). Restoration of Ylf proteins by reintroduction of *ylf* alleles onto the *L*. *pneumophila* chromosome (Fig 6B) complemented this defect in the efficiency of vacuole formation demonstrated by the Δ*ylfA* Δ*ylfB* mutant.

**Fig 7:**
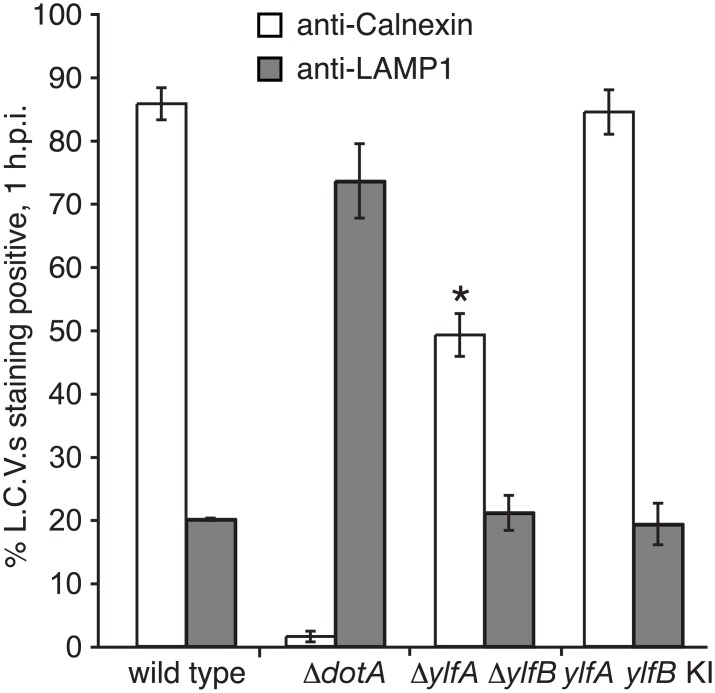
The Ylfs are required for efficient recruitment of ER-derived membrane to LCVs. PMA-differentiated U937 cells were infected with GFP-producing wild type, Δ*dotA*, Δ*ylfA* Δ*ylfB*, and *ylf* double knock-in strains of *L*. *pneumophila*. After 1 h of infection, *L*. *pneumophila*-containing vacuoles were recovered from the supernatant of infected cell lysates following low speed centrifugation. Vacuoles were stained with either anti-Calnexin or anti-LAMP1 antibodies to reveal interaction with host cell ER or endosomal compartments. Data represent the average of three independent experiments ± SD, n = 300 per condition, per experiment. Δ*ylfA* Δ*ylfB* mutant LCV Calnexin staining is significantly different compared to wild type in an unpaired Student’s *t* test.*p = 0.0001.

The reduced level of replicative vacuole formation by the Δ*ylfA* Δ*ylfB* mutant could result from a reduced ability of the mutant to either delay endocytic maturation or promote vacuole remodeling by recruitment of ER-derived vesicles, which are independent activities requiring Dot/Icm translocated effector proteins. To better define the Δ*ylfA* Δ*ylfB* mutant phenotype, the trafficking of wild type and mutant *L*. *pneumophila* strains were analyzed at 1 h post-infection. Consistent with previous studies [23], approximately 85% of LCVs isolated in a post-nuclear supernatant at 1 h post-infection stained positive for the ER protein Calnexin, indicating successful recruitment of membrane traffic from the host secretory pathway (Fig 7). A small population of wild type bacteria (~20%) was found to reside in vacuoles that stained positive for the lysosomal protein LAMP1, indicative of the fraction of wild type bacteria that did not successfully evade endocytic maturation. Conversely, infection by Δ*dotA* mutant *L*. *pneumophila*, which lack a functional Dot/Icm secretion system, resulted in minimal levels of anti-Calnexin staining (~2%) and substantial anti-LAMP1 colocalization (~74%). A lower percentage of Δ*ylfA* Δ*ylfB* mutant-containing vacuoles were Calnexin positive (50%) compared with vacuoles containing wild type *L*. *pneumophila* (85%), which is consistent with the 30% decrease observed in the efficiency of the Δ*ylfA* Δ*ylfB* mutant strain in creating vacuoles that support replication. Importantly, the percentage of Δ*ylfA* Δ*ylfB* mutant vacuoles that stained positive for LAMP1 was not significantly higher at 1 h compared with vacuoles containing wild type *L*. *pneumophila*. Thus, the YlfA and YlfB proteins do not appear to be involved in the ability of *L*. *pneumophila* to interfere with rapid endocytic maturation events. Taken together, these data suggest that the decreased efficiency of LCV formation by the Δ*ylfA* Δ*ylfB* mutant is due primarily to a defect in the recruitment of host membranes from the early secretory pathway.

## Discussion

*Legionella pneumophila* survives and replicates within eukaryotic cells by altering the transport and composition of the vacuole in which it resides [37]. The bacterial proteins required to modulate vacuole transport and biogenesis are translocated into host cells by the Dot/Icm secretion system [9,10,23,24,38]. In recent years, numerous studies have yielded a long list of putative and confirmed Dot/Icm substrates, however, the function of most of these proteins remains unknown [19,20]. Here we investigated the biochemical properties of two Dot/Icm substrates, the paralogs YlfA and YlfB, in an effort to determine their contribution to the intracellular lifecycle of *L*. *pneumophila*.

Both Ylf proteins have a region of hydrophobic residues near the amino terminus followed by two domains predicted to form coiled-coil helices. Using fluorescence microscopy and cellular fractionation, we have shown that the hydrophobic amino-termini of YlfA is able to localizing this protein to eukaryotic cell membranes. On the other hand, for YlfB our data suggests that membrane localization is driven by the predicted coiled-coil domains of the protein. Interestingly, we find a discrepancy in the localization of YlfB in the two approaches used. On the one hand, we can see membrane localization in the construct that lacks the amino terminus hydrophobic region, when using Immunofluorescence studies. On the other hand, in fractionation experiments, a similar construct localizes exclusively in the soluble fraction. We speculate that there may be two distinct mechanisms driving the membrane localization of YlfB in eukaryotic cells. One mechanism requires the hydrophobic region, and seems to be stable during fractionation, and the other mechanism requires the predicted coiled coil domains of the protein but may be unstable and lost during the fractionation process, yet is retained during immunofluorescence. The specific localization of these ectopically produced proteins to the membranes of the ER and early secretory pathway cannot be directly interpreted as an indication of their targeting during infection. However, previous studies have shown that at late time points, translocated YlfA localizes to membranes associated with the LCV and also to punctate structures dispersed throughout the infected cell [27]. These punctae largely colocalize with anti-KDEL staining, indicating that a subpopulation of YlfA translocated during infection associates with a population of vesicles derived from the early secretory pathway. Taken together, these data indicate that upon translocation into host cells, YlfA and YlfB associate with the membranes of the ER and early secretory network, and this association is mediated by the hydrophobic residues in the Ylf amino termini.

*In silico* screening of the *L*. *pneumophila* genome has revealed a large number of Dot/Icm substrates that contain protein-protein interaction motifs such as coiled coils, leucine rich repeats and ankyrin repeat homology domains [28,39]. Indeed, it is the very presence of these domains that has marked these proteins as putative effectors, based on their presumed ability to interact with, and therefore modulate the function of the host proteins these domains target. However, the hypothesis that these eukaryotic-like interaction motifs exist exclusively to target Dot/Icm substrates to host factors may be too simplistic. Here, we demonstrate that the coiled coil domains of YlfA and YlfB serve to mediate binding of these proteins in homomeric and heteromeric complexes, indicating that eukaryotic-like protein binding motifs on *L*. *pneumophila* effectors can serve as homotypic interaction domains or platforms for binding other effectors. Though a search for additional Ylf binding partners within the host cell has not been exhaustive, attempts to isolate *bone fide* host targets whose function is modulated by a Ylf protein have so far been unsuccessful [40]. Thus, our findings suggest that in addition to translocating effectors that have very specific host targets, such as LepB and DrrA/SidM, *L*. *pneumophila* may also utilize effectors with biochemical activities that do not involve the specific targeting of host proteins. Our findings emphasize the potential importance of effector-effector interaction inside of the host cell. Effector proteins can also function in two modalities, by interacting with both a host factor and another effector protein, as was shown for the *L*. *pneumophila* effector LubX, which targets the effector SidH to proteasome-mediated degradation in the host cell [41].

Previous studies of *L*. *pneumophila* mutants lacking YlfA and YlfB failed to detect a replication defect for single or double mutants using a CFU-based analysis of intracellular replication [27]. A more sensitive single-cell assay, however, has revealed a 30% decrease in the ability of Δ*ylfA* Δ*ylfB* mutant to establish vacuoles that support replication relative to wild type *L*. *pneumophila*. By quantifying the acquisition of ER or endocytic markers, it was determined that LCVs containing the Δ*ylfA* Δ*ylfB* mutant were capable of evading fusion with endocytic compartments, but had a reduced capacity to recruit membrane traffic from the ER. Both LCV formation and phagosome remodeling defects seen in double mutant infections could be complemented by reintroduction of the *ylf* alleles. It is yet to be determined whether the closely related Ylf paralogs contribute distinct activities, or may function in a redundant fashion. The latter possibility would be consistent with their ability to interact with themselves and each other using the same set of coiled coil domains. Overall, these data implicate the Ylf proteins as being involved in early events that occur during biogenesis of vacuoles that support *L*. *pneumophila* replication.

While these observations do not provide direct evidence of the function of YlfA and YlfB, our data indicate that these proteins are involved in the acquisition of ER-derived membrane by the LCV. The sequence similarity shared by YlfA and the *Chlamydophila pneumonia* IncA is intriguing, due to the ability of IncA to bind and possibly recruit host SNARE proteins to the bacterial inclusion [32]. Modeling of IncA has revealed significant structural similarity with SNARE motifs found in eukaryotic membrane fusion proteins [32,42]. Given these findings, the resistance of Ylf homodimers to dissociation with SDS and boiling is of particular interest, as stability under such conditions is one of the hallmarks of SNARE protein complexes. When produced in yeast, YlfA and YlfB formed large structures around the yeast vacuole, and the production of YlfA resulted in a vacuolar-sorting defect [43]. However, the Ylf proteins did not seem to inhibit the homotypic fusion of yeast vacuoles in vitro, suggesting that the Ylf proteins do not interact specifically with the yeast fusion machinery [44]. Whether the Ylf proteins serve as homemade autonomous fusion machinery used to combine ER-derived vesicles with the *L*. *pneumophila* -containing vacuole is an interesting possibility that is worthy of further investigation.

## Materials and Methods

### Bacterial Strains and Media

All bacterial strains, plasmids and oligonucleotide primers used in this study are listed in Table 1. *L*. *pneumophila* strains were grown on charcoal-yeast extract (CYE) plates or in ACES-buffered yeast extract (AYE) broth as described previously [45]. As required, *L*. *pneumophila* media was supplemented as follows: 100 μg ml^-1^ streptomycin, 20 μg ml^-1^ kanamycin, 6.25 μg ml^-1^ chloramphenicol, 0.1 mM IPTG, 5% sucrose. *Escherichia coli* strains were grown on L-agar plates or in L-broth, and supplemented with antibiotics as required: 100 μg ml^-1^ ampicillin, 40 μg ml^-1^ kanamycin, and 25 μg ml^-1^ chloramphenicol.

**Table 1:** Strains, plasmids and primers.

**STRAINS:**			
**Name**	**Genotype**		**Reference**
***L*. *pneumophila***			
CR39	*L*. *pneumophila* serogroup 1, strain LP01 *rpsL*		[14]
CR58	*dotA rpsL*		[51]
CR1572	*ylfA rpsL*		[27]
CR1576	*ylfB rpsL*		[27]
CR1578	*ylfA ylfB rpsL*		[27]
CR1698	*ylfA ylfB rpsL*:: *ylfA*		This study
CR1699	*ylfA ylfB rpsL*:: *ylfB*		This study
CR1700	*ylfA ylfB rpsL*:: *ylfA*:: *ylfB*		This study
CR1702	*ylfA ylfB rpsL* pAM239		This study
CR1702	*ylfA ylfB rpsL*:: ylfA pAM239		This study
CR1703	*ylfA ylfB rpsL*:: ylfB pAM239		This study
CR1704	*ylfA ylfB rpsL*:: *ylfA*:: *ylfB* pAM239		This study
CR1705	*ylfA ylfB rpsL* pEMC124		This study
CR1714	*ylfA ylfB rpsL* pEMC103		This study
***E*. *coli***			
DH5			Gibco BRL
CR014	DH5 *pir*		[51]
BL21 (DE3)			Stratagene
**PLASMIDS:**			
**Name**	**Relevant properties**	**Selection**	**Reference**
pEMC11	pCDNA3 YlfA	Amp	[27]
pEMC12	pCDNA3 YlfA (122–425)	Amp	[27]
pEMC175	pCDNA3 YlfB	Amp	This study
pEMC179	pCDNA3 YlfB (130–405)	Amp	This study
pEMC14	pEGFP C2 YlfA	Kan	[27]
pEMC93	pEGFP C2 YlfA (122–425)	Kan	This study
pEMC99	pEGFP C2 YlfB	Kan	This study
pEMC102	pEGFP C2 YlfB (124–405)	Kan	This study
pEMC109	pmRFP C1 YlfA	Kan	This study
pEMC111	pmRFP C1 YlfA (163–425)	Kan	This study
pEMC17	pGEX-KG YlfA (128–425)	Amp	[27]
pEMC144	pGEX-KG YlfB (131–405)	Amp	This study
pEMC124	pMMB207M45- YlfA	CM	This study
pEMC135	pmRFP C1 YlfB (301–405)	Kan	This study
pEMC136	pmRFP C1 YlfB (1–298)	Kan	This study
pEMC187	pSR47S ylfB insertion construct	Kan	This study
pSR47S	Gene replacement vector	Kan	[52]
pCDNA3	eukaroytic expression vector	Amp	Invitrogen
pEGFP C2	N-terminal EGFP fusion vector	Kan	Clonetech
pmRFP C1	N-terminal mRFP fusion vector	Kan	Clonetech
pMMB207M45	N-terminal M45 fusion vector	CM	[53]
pMMB207M45-	pMMB207M45 without XbaI	CM	This study
pGEX-KG	GST fusion vector	Amp	[54]
pAM239	Legionella GFP vector	CM	[53]
**PRIMERS:**			
**Name**	**Restriction Site**	**Sequence**	
EMC1	XbaI	AACTCTAGACCAGTTTTATTAAACGATTATGG	
EMC4	SacI	AGCGAGCTCGATAGAAACTTGTCTGCCATC	
EMC10	SalI	GGGTCGACCTTAATTGACTAAAGCAATAG	
EMC12	EcoRI	CCGAATTCACAGGACTAACCTATACCGCAG	
EMC17	SacI	GGGAGCTCGATACCCATATCATGCTTGG	
EMC20	XbaI	GGTCTAGACCGTATTGCCTATTATATACG	
EMC71	EcoRI	CCGGGAATTCATGACAGACACTCCAAAAGCTAA	
EMC72	XhoI	GGCCCTCGAGCTAACCTGTGAGAGTTTGAGTTG	
EMC75	SalI	GGGTCGACCTAACCTGTGAGAGTTTGAG	
EMC76	EcoRI	CCGAATTCGAGCATATTACTGATCG	
EMC79	PstI	TTCTGCAGCTAACCTGTGAGAGTTTGAGTTGGG	
EMC84	BamHI	CGCGGATCCGGATGACAGACACTCCAAAAGCT	
EMC93	HindIII	GGAAGCTTCTAACCTGTGAGAGTTTGAG	
EMC98	EcoRI	CCGGAATTCTAATGACAGACACTCCAAAAGC	
EMC103	SacI	CCGAGCTCAAATGGCTACTAATGAAAC	
EMC104	SacI	CCGAGCTCAAGAGGCATTGGATGC	
EMC105	KpnI	CCGGTACCTTAATTGACTAAAGC	
EMC123	SalI	CCGTCGACGATGGCTACTAATGAAAC	
EMC126	PstI	GGCTGCAGTTAATTGACTAAAGC	
EMC173	EcoRI	GCGAATTCCGCCACCATGGAAGAGCATATTACTGATCG	
EMC175	EcoRI	CCGGGAATTCCCCGCCACCATGACAGACACTCCAAAAG CTAA	

### Cell Culture

Chinese hamster ovary cells expressing FcγRII [24] were maintained in minimal Eagle’s medium α supplemented with 10% heat-inactivated fetal bovine serum (FBS-HI). HEK293T cells (ATCC no. CRL-11268) were maintained in Dulbecco modified Eagle’s medium supplemented with 10% FBS-HI. U937 cells (ATCC no. CRL-1593) were maintained and differentiated in RPMI 1640 medium containing l-glutamine and supplemented with 10% FBS-HI. 48 h prior to infection, differentiation was induced with 10 ng ml^-1^ phorbol 12-myristate 13-acetate (PMA) (Sigma no. P1585), and cells were lifted and replated 12 hours prior to infection. Bone marrow-derived macrophages were cultured from female A/J mice (Jackson Laboratory) as described previously [46]. Unless otherwise noted, all culture media and supplements were obtained from Gibco BRL.

### Plasmid and Strain Construction

For all plasmid construction, CR39 (wild type) genomic DNA was used as a template for PCR. Plasmids expressing full length and truncated YlfB were constructed using a common reverse primer, EMC72, and forward primers EMC175 and EMC173 to generate PCR products encoding YlfB 1–405 and YlfB 130–405, respectively. PCR products were digested with EcoRI and SalI and ligated into pCDNA3 digested with the same enzymes to generate plasmids pEMC175 and pEMC179.

For the plasmid expressing GFP-YlfA 122–425, the forward primer EMC12 and reverse primer EMC10 were used to generate a PCR product encoding YlfA 122–425. For GFP-YlfB plasmids, a common reverse primer, EMC75, and forward primers EMC71 and EMC76 were used to generate PCR products encoding YlfB 1–405 and YlfB 124–405, respectively. PCR products were digested with EcoRI and SalI and ligated into pEGFP C2 digested with the same enzymes to generate plasmids pEMC93, pEMC99 and pEMC102.

For plasmids expressing RFP-YlfA fusion proteins, a common reverse primer, EMC109, and forward primers EMC103 and EMC104 were used to generate PCR products encoding YlfA 1–425 and YlfA 163–425, respectively. PCR products were digested with SacI and KpnI and ligated into pmRFP C1 digested with the same enzymes to generate plasmids pEMC109 and pEMC111.

For the plasmid expressing GST-YlfB 131–405, the forward primer EMC98 and the reverse primer EMC93 were used to generate a PCR product encoding YlfB 131–405. Ligation of this PCR product and pGEX-KG digested with EcoRI and HindIII generated the plasmid pEMC144.

For the plasmid expressing M45-YlfA, the forward primer EMC123 and reverse primer EMC126 were used to generate a PCR product encoding YlfA 1–425. pMMB207M45 was modified to eliminate the XbaI site by digestion with BamHI and XbaI, blunt ending with Klenow, and religation with T4 DNA ligase. This plasmid (pMMB207M45-) and the PCR product were digested with SalI and PstI and their ligation generated the plasmid pEMC124. To generate a plasmid producing M45-YlfB, the forward primer EMC84 and reverse primer EMC79 were used to generate a PCR product encoding YlfB 1–405. This product and pMMB207M45 were digested with BamHI and PstI and ligated to generate the plasmid pEM103.

To restore a complete *ylfA* open reading frame to the Δ*ylfA* Δ*ylfB* mutant *L*. *pneumophila* genome, the forward primer EMC4 and reverse primer EMC1 were used to generate a PCR fragment encoding YlfA 1–425 as well as approx. 300 bp of flanking 5’ and 3’ sequence. This PCR product and the suicide vector pSR47S were digested with SacI and XbaI and ligation generated the plasmid pEMC186. The *ylfA* ORF was introduced onto the chromosome of CR1578 by allelic exchange. First, the insertion plasmid was mated with *E*. *coli* onto CR1578 as described previously. Mating mixtures were plated on CYE containing kanamycin and streptomycin to select for *L*. *pneumophila* that had integrated the insertion plasmid onto the chromosome. Kanamycin-resistant colonies were then plated on CYE containing 5% sucrose to select for bacteria that had lost the plasmid. Sucrose-resistant colonies were screened by single colony PCR to identify clones that had incorporated the full-length allele. A similar strategy was used to restore the *ylfB* open reading frame, with the complementation plasmid pEMC187 contains an insertion fragment generated by PCR using forward primer EMC17 and reverse primer EMC20. The double complemented strain was generated by introduction of *ylfB*, followed by *ylfA* onto the CR1578 chromosome.

### Fluorescence Microscopy

To localize ectopically produced GFP-Ylf fusion proteins, CHO cells were transfected with plasmids pEMC14, pEMC93, pEMC99 and pEMC102 using the Fugene6 transfection reagent (Roche). 18 h after transfection, cells were fixed with 2% paraformaldehyde (PFA) for 20 min, permeabilized with cold methanol and blocked with PBS containing 2% goat serum and 50 mM NH_4_Cl for 1 h. Coverslips were incubated in mouse anti-KDEL (Santa Cruz) (1:250), followed by an Alexa594-conjugated goat anti-mouse secondary (Invitrogen) (1:500). Localization of co-produced YlfA and YlfB was tested by cotransfection of CHO cells with plasmids pEMC109 and pEMC99. Images were collected using a Zeiss LSM 510 laser scanning confocal microscope to capture single 0.2 μm Z-sections, which were exported as TIFF files and labeled in Adobe Illustrator.

### Cellular Fractionation

HEK293T cells were transfected with plasmids pEMC11, pEMC12, pEMC175 and pEMC179. 14 h after transfection, cells were harvested and suspended in lysis buffer (0.25 M sucrose 10 mM Tris-HCl pH 7.4) containing Complete protease inhibitor cocktail (Roche), and lysed by ball bearing homogenization. Unlysed cells were removed by centrifugation at 200 x g for 3 min. For conventional fractionation, soluble and membrane fractions were separated at 100,000 x g for 1 h at 4°C. Membrane pellets were resuspended in lysis buffer and solubilized in 1% Triton X-100. Triton X-100 soluble and insoluble components were separated by a second spin as described above. For density gradient analysis, 1 ml of lysate was floated on top of a 10 ml 4–26% OptiPrep (Axis-Shield PoC AS) gradient and centrifuged at 100,000 x g for 3 h at 4SC. 0.5 ml fractions were collected from the top of the gradient. Fraction samples were separated on 12% SDS-PAGE and Ylf proteins were detected by immunoblot using rabbit polyclonal anti-YlfA and anti-YlfB antibodies (1:5000). Fractionation efficiency was monitored by detection of the ER integral membrane protein Calnexin and soluble α-tubulin with rabbit anti-Calnexin (Assay Designs) (1:2000) and rat anti-α tubulin (Accurate Chemical & Scientific Corp.) (1:1000). Primary antibodies were detected with HRP-conjugated goat anti-rabbit and donkey anti-rat secondary antibodies (Zymed) (1:2000).

### Ylf Protein Binding Analysis

Logarithmic cultures of BL21 (DE3) *E*. *coli* strains containing plasmids encoding GST, GST-Ylf 128–425 or GST-YlfB 131–405, and M45-YlfA or M45-YlfB were induced with 1mM IPTG for 6 h and lysed by French press. Cellular debris was cleared by centrifugation at 20,000 x g for 20 min and incubated with glutathione sepharose 4B resin for 2 h. After washes in PBS, PBS 10% glycerol, PBS 1M NaCl and PBS 3M NaCl, bound M45-tagged proteins were eluted in 20 mM reduced glutathione and detected by anti-M45 immunoblot. Total eluted protein was detected by staining of PVDF membranes with Ponçeau S.

GST-tagged proteins were purified from DH5α *E*. *coli* strains containing plasmids encoding GST, GST-YlfA 128–425 and GST-YlfB 131–405 as previously described [27]. For far Western blot analysis, liquid cultures of *L*. *pneumophila* strains CR39, CR1572, CR1576, CR1578, CR1702 and CR1703 were to stationary phase and harvested by centrifugation at 5,000 x g for 10 min and resuspended in cold PBS. Cells were lysed by sonication and cleared of debris by centrifugation at 20,000 x g for 10 min. Lysates were combined with Laemmli sample buffer, boiled and separated on 10% SDS-PAGE. After transfer to PVDF, membranes were blocked in PBS containing 5% NFDM and 0.1% Tween 20 and incubated overnight at 4°C in blocking solution containing 20 μg ml^-1^ recombinant purified GST, GST-YlfA 128–425 or GST-YlfB 131–405. Membranes were washed in blocking solution and bound protein was detected with a primary monoclonal anti-GST antibody [47] (1:1,000) and a secondary HRP-conjugated anti-mouse IgG antibody (Zymed) (1:2,000).

### Gel Filtration

Recombinant purified 6xHis-YlfA 128–425 and 6xHis-YlfB 131–405 were suspended at 1 μg ml^-1^ in 10% glycerol PBS, centrifuged at 100,000 x g for 1 h, and applied to a Superdex 200 column. Fractions were monitored by UV absorbance and anti-YlfA and anti-YlfB immunoblot. To test for stability of high molecular weight structures, samples were combined with Laemmli buffer containing β-mercaptoethanol and 0.1% SDS, and boiled for 10 min.

### Replicative Vacuole Assay

Bone marrow-derived macrophages (BMM) were plated at 1 x 10^5^ cells per coverslip and infected at an MOI of 20 with *L*. *pneumophila* strains grown for 48 h to early stationary phase on CYE. Plates were centrifuged at 200 x g for 5 min to initiate contact and synchronize infection. 2 h post-infection, uninternalized bacteria were removed by washing with warm PBS. One set of coverslips was fixed in 2% PFA, and fresh media was added to the remaining samples for an additional 8 h of incubation. Following fixation, samples were blocked and external/adherent bacteria were detected using an anti-*L*. *pneumophila* antibody (1:1000), followed by an AlexaFluor594-conjugated chicken anti-rabbit secondary (Invitrogen) (1:500). Following permeabilization of cells with cold methanol, all bacteria were detected using the anti-*L*. *pneumophila* antibody, and an AlexaFlour488-conjugated goat anti-rabbit secondary (Invitrogen) (1:500). BMM and bacterial DNA were stained with 0.1 μg ml^-1^ DAPI. To determine the efficiency of replicative vacuole formation, the number of BMM containing a replicative vacuole (3+ bacteria) at 10 h post-infection was divided by the number of infected BMM at 2 h post-infection.

### Bacterial Vacuole Isolation and Analysis

Procedures for infection of differentiated U937 cells and analysis of isolated LCVs were adapted from those described previously [23]. 1 x 10^7^ differentiated U937 cells were infected at an MOI of 5 with *L*. *pneumophila* strains grown for 48 h to early stationary phase on CYE. 1 h post-infection, cells were placed on ice and uninternalized bacteria were removed by washing with cold PBS. Infected cells were harvested with a rubber policeman, pelleted and respuspended in lysis buffer (20 mM HEPES, 250 mM sucrose, 0.5 mM EGTA, pH adjusted to 7.2 with KOH). Cells were lysed by ball bearing homogenization and LCVs were recovered in post-nuclear supernatant (PNS) fractions following low speed centrifugation (200 x g for 3 min). PNS was added to poly-l-lysine coated coverslips and fixed with either 4% PFA for anti-Calnexin staining, or PLP-sucrose fixative for anti-LAMP1 staining. For anti-Calnexin staining, coverslips were incubated in block (PBS containing 2% goat serum and 50 mM NH_4_Cl), followed by anti-Calnexin (1:250) and an Alexa594-conjugated chicken anti-rabbit secondary (1:500). For LAMP1 detection, coverslips were blocked in PBS containing 2% BSA and 0.1% Saponin, followed by incubation with anti-LAMP1 (H4A3) (1:50) and an Alexa594-conjugated goat anti-mouse secondary (1:500), with all washes and antibody incubations performed using block solution.

## Supporting Information

S1 Fig Cellular localization of RFP-tagged YlfB protein truncations. CHO cells were transfected with plasmids encoding amino-terminal RFP-tagged proteins: YlfB 1–298 and YlfB 301–405. 18 h after transfection, cells were fixed and imaged.
(EPS)
